# Primary lymphoma of the colon: report of two cases and review of literature

**DOI:** 10.1186/s12957-018-1548-6

**Published:** 2019-01-15

**Authors:** Manoj Pandey, Jyoti Swain, Hema Malini Iyer, Mridula Shukla

**Affiliations:** 1 Department of Surgical Oncology, Institute of Medical Sciences, Banaras Hindu University, Varanasi, 221 005 India; 2 Department of Histopathology, Lal Path Labs, New Delhi, India; 3 Dharamsheela Cancer Centre, Delhi, India; 4 Department of Pathology, SRL Lab, Varanasi, India

**Keywords:** Lymphoma, Extranodal, Colon, Surgery, Chemotherapy, CD20, Immunohistohemistry

## Abstract

**Background:**

Gastrointestinal tract is the most frequent site of extranodal lymphoma accounting for approximately 40% of all extranodal lymphomas; out of these, non-Hodgkin’s lymphoma (NHL) comprises 4% of total cases. Primary lymphoma arising in the colon is very rare comprising only 0.2–1% of all colonic malignancy.

**Patients and methods:**

We report two cases of 13- and 20-year-old boys with NHL of colon presenting with abdominal pain and weight loss and discuss the approach to colonic lymphoma after a review of world literature to provide an overview on colonic lymphoma.

**Results:**

Colonic NHL most commonly affects older age group with mean age of diagnosis being 55 years. Abdominal pain and weight loss are the two most common presentations with palpable abdominal mass as physical examination finding in half of the cases.

**Conclusions:**

Colonic lymphoma in young adolescence is rare. Multimodality approach involving both surgery and chemotherapy is the principal mode of treatment. Radiotherapy is used in selected cases. If diagnosed preoperatively, non-surgical management can be attempted.

## Background

Primary colonic lymphoma is a rare entity comprising about 0.2–1% of all colonic malignancies^.^ [1–4]. Non-Hodgkin’s lymphoma (NHL) is the most common histological subtype. Males are most commonly affected with peak incidence in sixth and seventh decade of life [5]. Though lymphoma is common in children, extranodal primary colonic lymphoma is rare in childhood [6]. We report here two cases of colonic lymphoma in adolescent boys with a review of world literature.

## Case report

### Case 1

**Fig. 1:**
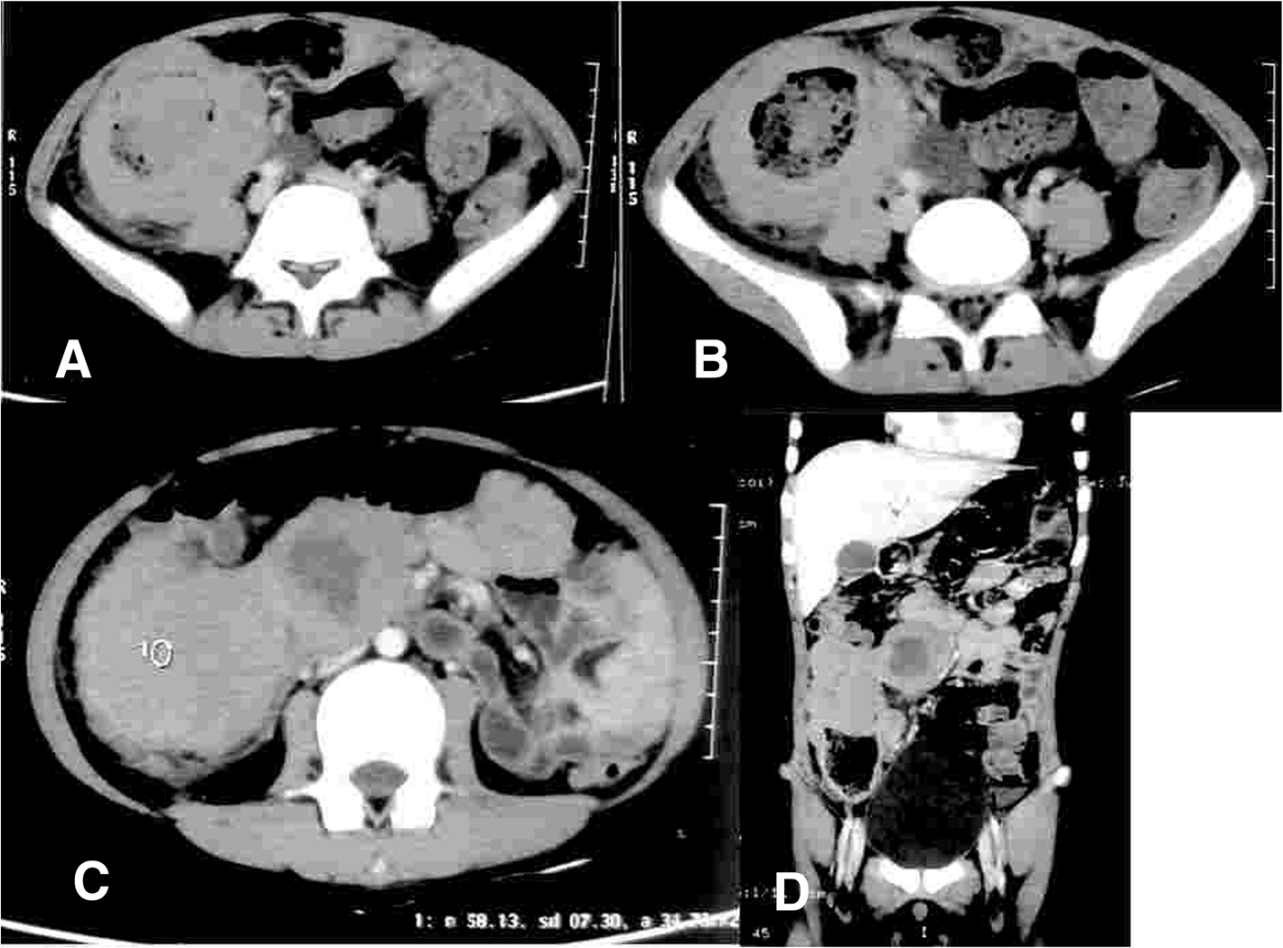
Computed tomography showing **a**) the lesion in the caecum, **b**) lesion in caecum with lymph nodes **c**) ascending colon and **d**) reaching up to transverse colon

A 13-year-old child presented with complaints of dull aching pain in the right lower abdomen since 3 months and lump in the right lower abdomen for 2 months. He also complained of intermittent vomiting with normal bladder and bowel habit. There was no associated fever and melena. Except for the presence of pallor, the general physical examination was normal. Abdominal examination revealed tender, hard 10 × 6-cm mass in the right iliac fossa extending to the midline. The mass had restricted intrinsic mobility, and it did not move with respiration. There was no organomegaly or any other findings on abdominal examination and digital rectal examination. Routine haematological and biochemical investigations were within normal limit except the haemoglobin of 8 g/dL. There was no occult blood in stool, and chest X-ray was normal. Ultrasonography of the abdomen revealed a large 10.2 cm × 6.73-cm hypoechoic area with anechoic component surrounded by gut loops and omentum in the right iliac fossa. Contrast-enhanced computed tomography scan of the abdomen revealed gross circumferential wall thickening of caecum and ascending colon with partial luminal obliteration. The average radial wall thickness was 4.3 cm, and the approximate length of thickened segment was about 10.8 cm (Fig. 1). Few mesenteric lymph nodes were seen in the right iliac fossa with largest one measuring 39 mm × 42 mm.

**Fig. 2:**
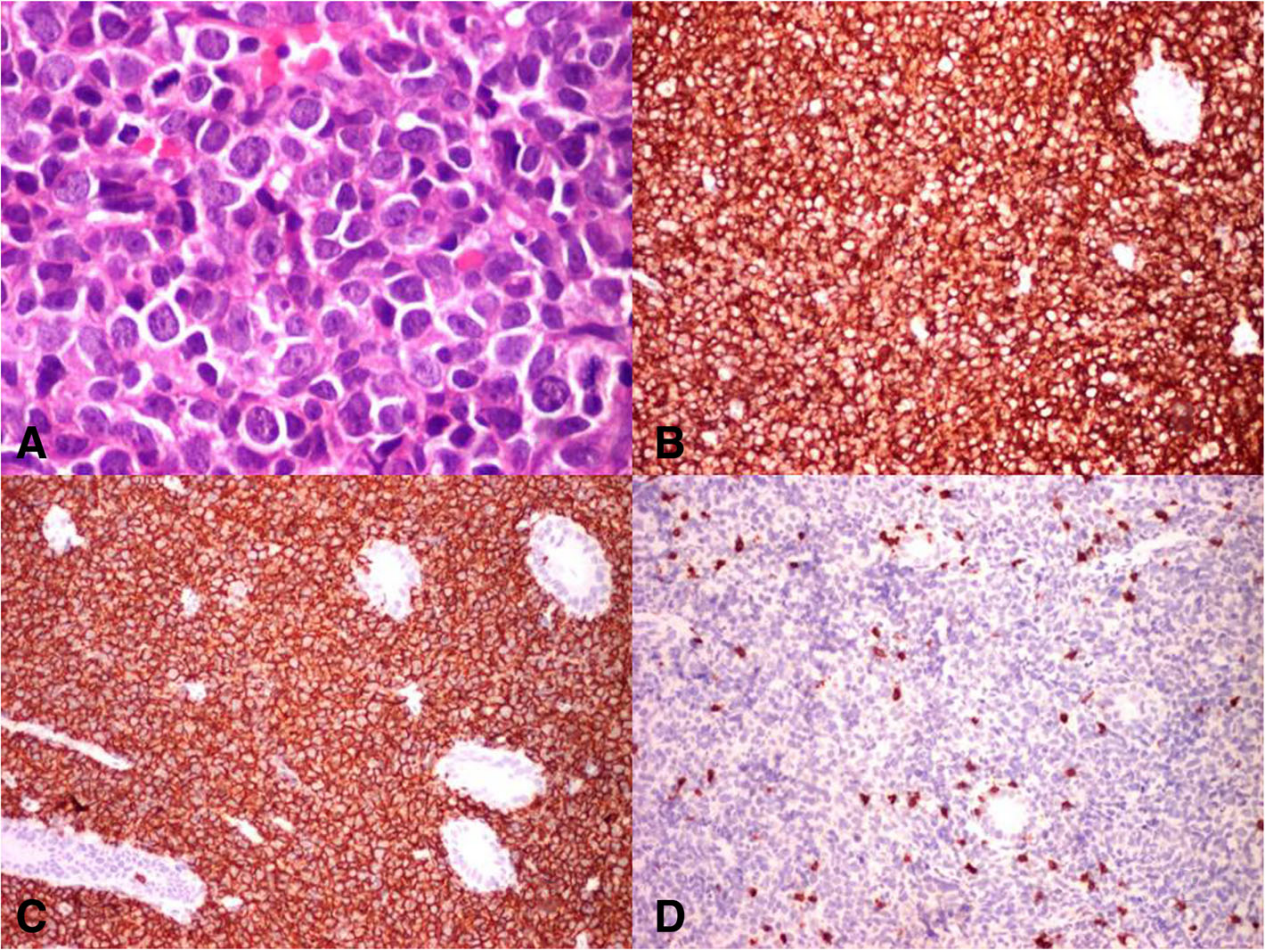
Photomicrograph showing **a** non-Hodgkin’s lymphoma H&E × 40, **b** diffuse CD45 positivity, **c** CD20 positivity, and **d** negative for CD3

A colonoscopy was performed that revealed ulceroproliferative mass in caecum extending up to ascending colon and a biopsy was taken. Histopathological examination of the biopsy revealed mucosal ulceration and necrosis with lamina propria showing sheets of monomorphic round cells having vesicular nuclei with distinct nucleoli which are strongly suggestive of non-Hodgkin’s lymphoma (Fig. 2). Immunohistochemistry showed CD20 and CD45 positivity while CD3 and cytokeratin were negative (Fig. 2). With the diagnosis of diffuse large B-cell lymphoma (DLBCL), the patient was started on R-CHOP (rituximab, cyclophosphamide, vincristine, and prednisolone). The patient is now doing well and is on regular follow-up.

### Case 2

A 20-year-old boy presented with complaints of altered bowel habits and abdominal pain of 6 months duration. The general physical examination and abdominal examination including digital rectal examination were normal. Haematological and biochemical parameters were remarkable, and there was no occult blood in faeces.

**Fig. 3:**
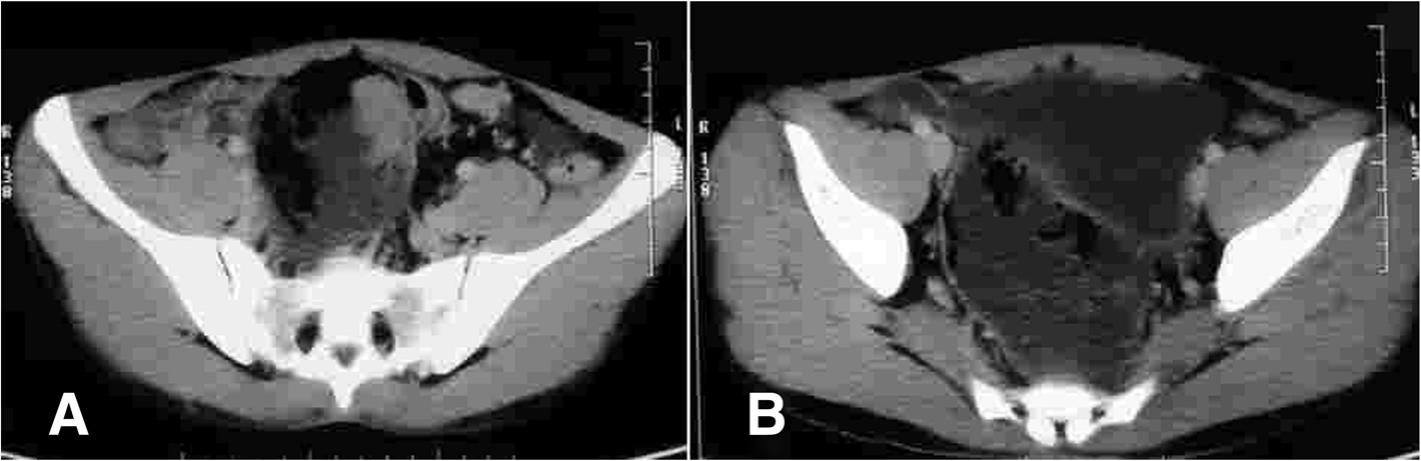
Computed tomographic images showing **a**) tumor at rectosigmaoid junction **b**) lesion in upper rectum

**Fig. 4:**
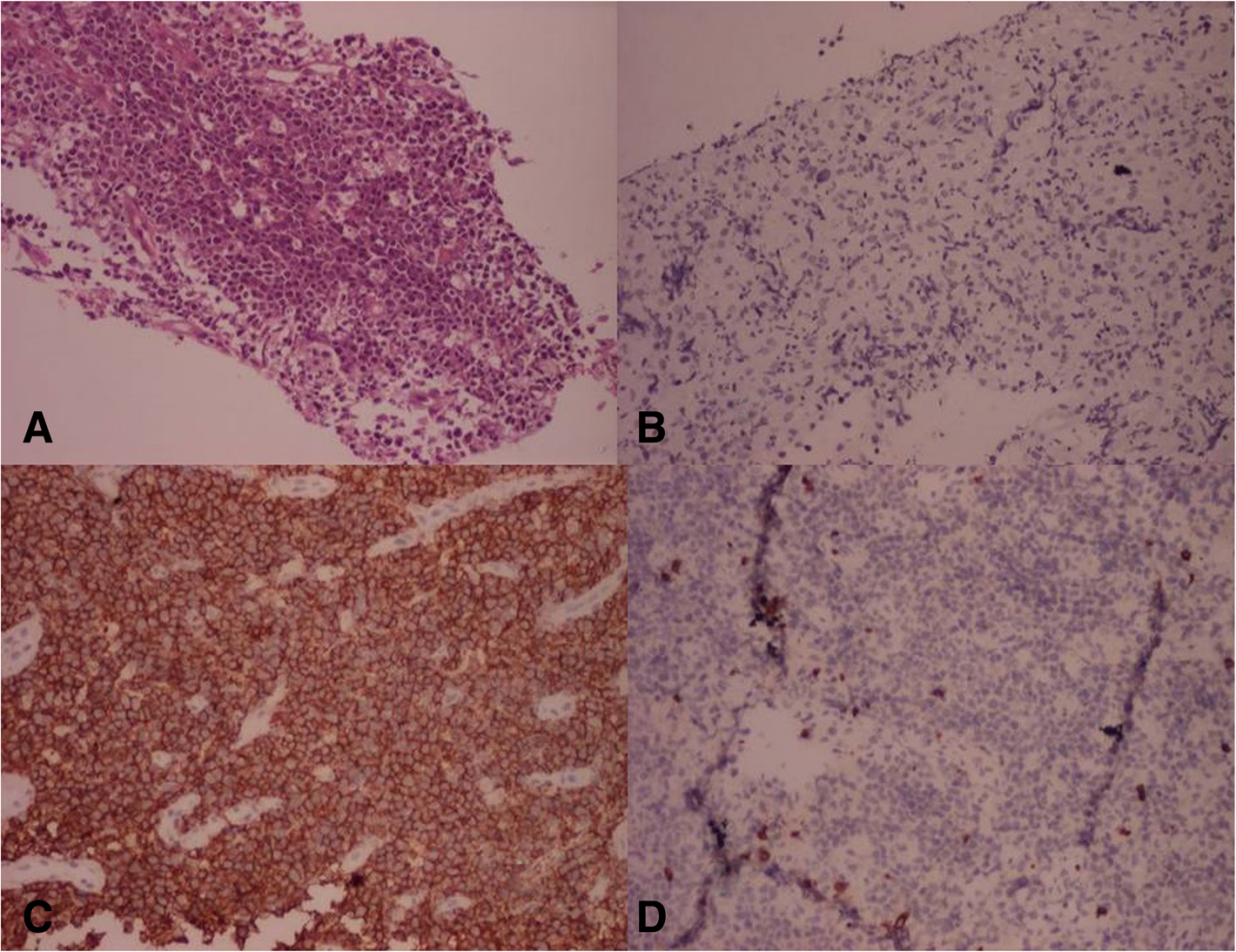
Photomicrograph showing **a** sheets of monomorphic round cells having vesicular nuclei with distinct nucleoli H&E × 10, **b** cytokeratin negative, **c** CD20 positivity, and **d** CD 3 negative

A CT scan was carried out that showed a mass in the pelvis arising from rectosigmoid junction; the planes with the bladder were well maintained, and there was no lymphadenopathy or liver/splenic lesions (Fig. 3). The patient underwent a colonoscopic examination that showed a proliferative growth in the upper rectum and rectosigmoid junction about 15 cm from anal verge. A biopsy was taken that revealed it to be lymphoma. Immunohistochemistry was performed, and tumour cells were positive for CD20 and CD45 while CD3 was negative (Fig. 4). A diagnosis of diffuse large B cell lymphoma was made.

Patient was started on R-CHOP chemotherapy and had a complete response. Patient is on regular follow-up and is disease-free after 2 years.

## Discussion

Gastrointestinal lymphoma accounts for 5–10% of all non-Hodgkin’s lymphoma with intestinal lymphoma contributing 15–20% of all gastrointestinal lymphoma [1]. Majority of these arise in the stomach (up to 65% of all GI lymphoma) followed by the small bowel (20–30%) with rest arising in the colon and rectum [1–3, 5]. B cell lymphoma of the colon is the third commonest malignancy of the colon after carcinoma and carcinoid though its incidence is less than 0.5%. Primary gastrointestinal malignancies are very rare in children with non-Hodgkin’s lymphoma being the most common [7]. Within the colon, the involvement of caecum is the commonest, and Gonzalez et al. [5] found caecum (60%) to be the commonest site, followed by the right (27%) and the sigmoid colon (13%). Bairey et al. [3] in their case series of 17 patients also found ileocaecal region to be the most common site of colonic lymphoma accounting for 76% of cases. In our case reports, the site of involvement was also caecum and ascending colon in one and rectosigmoid junction in the second.

According to the World Health Organization (WHO) classification, B cell lymphomas are classified into diffuse large B cell lymphoma, extranodal marginal zone lymphoma (mucosa-associated lymphoid tissue [MALT]-associated lymphoma), mantle cell lymphoma (MCL), Burkitt’s lymphoma, and follicular lymphoma [8]. Diffuse large cell B lymphoma (DLBCL) is the most frequent histological subtype affecting the gastrointestinal tract and colon [9]. DLBCL are composed of rapidly proliferating cells and are more aggressive than other B cell lymphomas.

Primary colorectal lymphoma mainly affects older age group in the fifth to seventh decade of life with male:female ratio being 1.5:1 [10]. Wong and Eu [11] in their study of 14 cases found mean age of presentation to be 61 years, whereas Bairey et al. [3] found it to be 72 years. In contrast to the above studies, our patient presented with lymphoma at 13 and 20 years of age probably the youngest reported case in the literature.

The presentation of B cell lymphoma is varied, and symptoms depend on the site of lesion. Most commonly, abdominal pain, weight loss, abdominal mass, and hematochezia, besides the features of obstruction like nausea, vomiting, change in bowel habits [1, 3], obstruction [1, 5], intussusceptions [12, 13], and acute peritonitis due to intestinal perforation [1, 3], are present. Fan et al. [10] in their series of 37 patients reported abdominal pain and weight loss in 62% and 43% of cases respectively, whereas Bairey et al. [3] reported abdominal pain and weight loss respectively in 56% and 29% of cases. Intestinal obstruction is a very rare presentation due to more pliable nature of colorectal lymphoma and the absence of desmoplastic response; however, symptoms of partial obstruction are more frequent. More than half of the patients present with bulky disease usually more than 5 cm [3, 10, 14]. Surprisingly, in most cases of colonic B cell lymphoma reported in the literature, the B symptoms are often absent and fever is very rarely a presenting symptom. This is probably due to anatomical location wherein bowel-related symptoms appear first.

The most common imaging modalities used to image colonic lymphoma are contrast-enhanced computed tomography (CECT) of the abdomen or double contrast barium enema (DCBE), both of which complement each other. CECT abdomen provides extraluminal information like tumour size, depth of invasion, and regional nodal involvement, whereas DCBE provides more information about mucosal changes and gross tumour morphology. It is to be noted that both these modalities are not able to differentiate colonic carcinoma from lymphoma and diagnosis has to be confirmed by colonoscopy and biopsy. Immunohistochemistry are required for sub-classification. Full haematology including a peripheral smear, biochemistry, chest X-ray, CT scans, and bone marrow biopsy are required to rule out systemic involvement and for staging the disease. Though there is no consensus on what is ideal investigation for staging, evidence is slowly emerging to suggest usefulness of PET-CT for extranodal lymphoma as well.

The most widely used staging system in clinical practice is the Lugano classification which is based on Ann Arbor system modified by Carbone et al. [15]. Stage I is involvement of a single nodal group or single extranodal site (IE). Stage II comprises of involvement of more than one nodal group on the same side of the diaphragm or single extranodal site and adjacent lymph nodes (IIE). In stage III, there is involvement of multiple nodal sites on both sides of the diaphragm, including extranodal sites (IIIE) or spleen (IIIS). Stage IV is involvement of bone marrow, central nervous system, or diffuse visceral involvement.

The principal modality of treatment is combined modality treatment that includes surgery and chemotherapy [16]. Early stage tumours are treated with surgery followed by polychemotherapy, and advanced stage tumours are treated with multidrug chemotherapy [1, 3, 17]. However, since the availability of rituximab, the CD20-positive B cell lymphomas can be treated with polychemotherapy combined with immunotherapy with complete and lasting response making the surgery redundant in these cases, as can be seen from the present case reports wherein surgery was avoided due to preoperative diagnosis and polychemoimmuno therapy.

Stage IE or IIE, disease confined to the colon or rectum with (IIE) or without (IE) regional lymph node involvement, had been treated with surgery followed by adjuvant chemotherapy [18]. In most series of published literature, surgery was followed by adjuvant multiagent chemotherapy (CHOP) which has led to improved outcomes [2, 18, 19]. Adjuvant chemotherapy has been shown to improve median survival from 36 to 53 months in a series of 15 patients (out of which 8 received adjuvant therapy) [20]. Similarly, Fan et al. [10] reported an improvement in median survival from 24 to 36 months. Aviles et al. [19] reported 83% 10-year overall survival in patients with stage IE treated with surgery followed by adjuvant chemotherapy. However, other studies found surgical resection of localised, primary colonic lymphoma to provide excellent local disease control [2, 5]. One of the reasons for avoiding primary chemotherapy is the fear of perforation of the bowel.

Surgery is the prime mode of therapy for palliation of pain and emergent conditions like obstruction, perforation, and bleeding. In a series of 43 patients reported by Cai et al. [21], 56% of the patients required emergency operation. Similarly, in a case series of 46 patients by Zhai et al. [22], 13 patients required emergency surgery.

Rapidly proliferating and aggressive advanced lymphoma is best treated with chemotherapy. The CHOP chemotherapeutic regimen (cyclophosphamide, doxorubicin, vincristine, and prednisone) is a mainstay of treatment for all moderate and high grade B cell lymphomas. Addition of rituximab to standard CHOP regimen has led to improvement of progression-free, event-free, disease-free, and overall survival [23]. With the advent of new monoclonal antibodies like rituximab, there is a shifting trend towards use of chemotherapy in all cases with surgery reserved mainly for palliation of emergent condition [24].

However, due to rarity of disease, no prospective randomised clinical trials have been designed yet to define the optimal therapy of primary colonic lymphoma. Treatment decisions mainly depend upon expert opinion and consensus made on the basis of level II/III and IV evidence that is in abundance (Table 1).

**Table 1:** Published studies on colorectal lymphoma

Author (year) [Ref.]	Number of cases	Histological type (WHO)	Treatment modality	Survival
Bairey et al. 2006 [3]	17	Diffuse large B cell lymphoma	Multimodality, 9 Chemotherapy, 6 Surgery, 2	Median, 44 months
Musallam et al. 2010 [26]	9	Diffuse large B cell lymphoma, 6 Burkitt lymphoma, 3	Multimodality, 5 Chemotherapy, 3 Surgery, 1	Median, 25 months
Wong and Eu 2006 [11]	14	Diffuse large B cell lymphoma	Multimodality, 11 Surgery, 3	5-year overall survival, 57%
Kim et al. 2005 [27]	95	B cell, 82.1% T cell, 17.9%	Multimodality, 57 Chemotherapy, 23 Surgery, 15	5-year overall survival, 55.2%
Pandey et al. 2002 [28]	8	Large cell, 3 Small cell, 1 Mix large and small, 3 Lymphoblastic, 1	Surgery with adjuvant therapy in all	66% 4 years
Auger et al. 1990 [29]	22	Diffuse histiocytic lymphoma	Multimodality, 14 Surgery, 8	N/A
Zighelboim and Larson 1994 [30]	15		Multimodality, 3 Chemotherapy, 12	5-year survival, 27%
Gonzalez et al. 2008 [5]	15		Multimodality, 12 Surgery, 3	Median survival, 60 months
Doolabh et al. 2000 [2]	7	Small non-cleaved, 5 Large cell, 2	Multimodality, 6 Surgery, 1	N/A
Cho et al. 1997 [31]	23		Multimodality, 14 Chemotherapy, 4 Surgery, 3	10-year survival, 61%
Fan et al. 2000 [10]	37	High grade, 29 Intermediate and low grade, 8	Multimodality, 22 Chemotherapy, 2 Surgery, 13	Median survival, 24 months
Stanojevic et al. 2008 [14]	24		Multimodality, 20 Surgery, 4	Median survival, 41.9%
Busch et al. 1994 [32]	19		Multimodality, 14 Chemotherapy, 4 Surgery, 1	Median survival, 45 months
Zinzani et al. 1997 [1]	32	High grade, 26 Low grade, 6	Multimodality, 22 Chemotherapy, 10	5-year overall survival, 59%
Zhou et al. 2011 [25]	32	B cell, 22 (DLBCL most common) T cell, 10	B cell, multimodality (22) T cell, surgery (8)	B cell DFS at 55 months, 88.2% T cell, 3 alive after 23 months
She et al. 2011 [33]	10	B cell, 8 Burkitt, 1 Mantle cell, 1	Surgery, 7 Chemotherapy, 3	Median survival Surgery, 17 months Chemotherapy, 13 months
Zhai et al. 2012 [22]	46	B cell origin, 35 T cell, 11		5-year survival, 64.2% Progression-free survival, 49.3%
Pascual et al. 2013 [34]	7	Diffuse large B cell lymphoma, 6 Follicular lymphoma, 1	Multimodality, 2 Chemotherapy, 2 Surgery, 3	N/A
Huang et al. 2013 [35]	52	Diffuse large B cell lymphoma, 64.4%	Multimodality, 43 Chemotherapy, 9	5-year survival, 71%
Tevlin et al. 2015 [36]	11	Diffuse large B cell lymphoma	Multimodality, 9 Chemotherapy, 1 Radiotherapy alone, 1	Median event-free survival, 10 months

Prognosis is often mixed with median survival of above 5 years reported in various series. Fan et al. [10] found stage to be the most important prognostic factors for survival. Others found histological grade to be the most important prognostic factor [3, 14, 21]. Urgency of surgical operation has also been reported as an important factor affecting survival [3, 14] along with histological subtype wherein B cell lymphomas have better prognosis than T cell phenotype [22, 25].

## Conclusion

Primary colonic lymphoma is a rare clinical entity presenting most commonly in older age group. The presentation is often nonspecific which leads to delayed diagnosis and advanced stage at presentation. Surgery followed by chemotherapy is the recommended treatment; however, in select cases, chemotherapy alone with inclusion of rituximab can be used. There is fear of perforation with use of systemic therapy, although in most series, chemotherapy-induced perforations have not been reported and fear appears to be unfounded. The reported prognosis of colonic lymphoma is poor and recurrences are common; however, with use of R-CHOP, improved survival is reported.
